# Salvage Operation Using the Free Latissimus Dorsi Muscle Flap for Necrosis of the Free Scapular Flap After Harvesting Both Flaps in a Chimeric Pattern

**Published:** 2017-08-18

**Authors:** Naohiro Ishii, Shigeki Sakai, Kazuo Kishi

**Affiliations:** Department of Plastic and Reconstructive Surgery, Keio University, Tokyo, Japan

**Keywords:** free flap, salvage, chimeric flap, perforator, microsurgery

## DESCRIPTION

We describe the case of a 39-year-old man with a diabetic ulcer on his left heel and proximal plantar surface. After debridement of the ulcer and bone abrasion, we planned reconstruction with a free scapular flap.

## QUESTIONS

1. What complications are associated with small skin perforators?
2. How is a free chimeric flap secured during salvage operation?
3. What has a free chimeric flap been used for to date?
4. In what other flaps can you apply the present technique?

## DISCUSSION

In free-flap surgery, small skin perforators can show strong spasm or become inadvertently injured during dissection, traction, or coagulation. However, it is sometimes difficult to decide whether the injured small perforator flaps can be used with the injured skin perforator despite waiting for recovery from the injury. Furthermore, there have been only few reports on salvage operation in patients with irreversible injured perforators besides harvesting an alternative flap from another donor site.^1^^,^^2^

**Figure 1:**
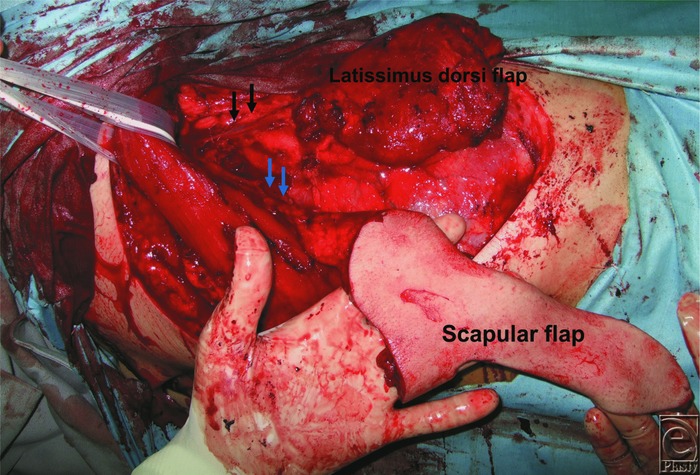
Harvesting flaps in a chimeric pattern. Blue arrow: the pedicle of the scapular flap is small. Black arrow: the pedicle of the latissimus dorsi muscle flap is intact.

**Figure 2:**
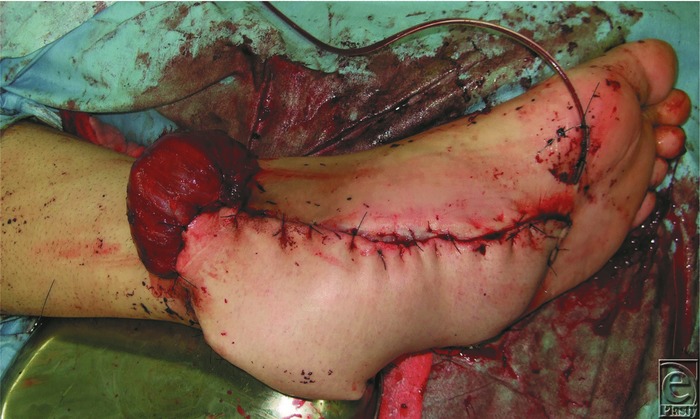
The setting of both flaps.

**Figure 3:**
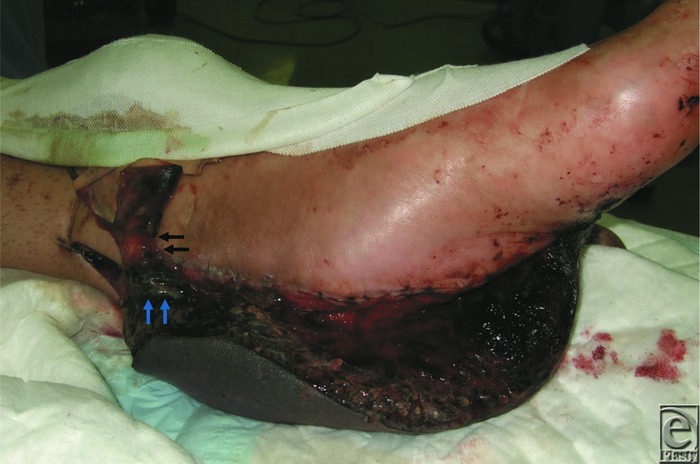
Postoperative circulation in both flaps. Blue arrow: the pedicle of the scapular flap has severe venous thrombosis. Black arrow: the pedicle of the latissimus dorsi muscle flap is intact.

**Figure 4:**
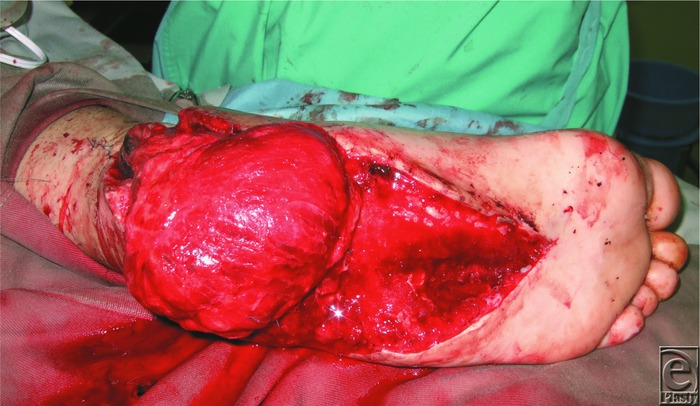
Salvage operation using the alternative flap. We decided to transfer the latissimus dorsi flap to the defects.

In harvesting a free scapular flap, the skin perforator of the scapular flap was small and showed strong spasm by traction and coagulation; furthermore, the flap pedicle still had weak pulsation despite using saline gauze with a vasodilator solution and waiting for 30 minutes. Subsequently, we dissected the latissimus dorsi flap and harvested both flaps in a chimeric pattern (Fig 1). The pedicles of both flaps, the subscapular artery and vein, were anastomosed smoothly in an end-to-end fashion with the left posterior tibial artery and great saphenous vein, respectively. The scapular flap may be usable, although it showed some venous congestion; therefore, it was sutured to the heel and plantar surface and the latissimus dorsi flap was sutured to the raw surface around the anastomosis as an alternative flap (Fig 2). Unfortunately, the scapular flap showed severe venous congestion and eventually failed on postoperative day 2. However, the latissimus dorsi flap had good circulation; therefore, reconstruction was successfully salvaged by the latissimus dorsi flap and skin graft (Figs 3 and 4). The course after salvage operation was uneventful, and the patient had a good outcome.

A chimeric flap is composed of different flaps, each supplied by different branches from the same source vessel; it was first introduced by Hallock.^3^ Flaps in a chimeric pattern are often used for multiple defects, even for implantable Doppler surrogate monitoring^4^; however, the use of an alternative flap has not been reported.

Our technique can also be applied for anterolateral thigh flaps and vastus lateralis or rectus femoris muscle flaps, or with latissimus dorsi myocutaneous flap and a serratus anterior muscle flap.

We believe that harvesting flaps in a chimeric pattern and delaying the decision about which flaps can be used, as in our case, may be a useful option to avoid other donor site violation.
